# Defining long-term survivors in metastatic lung cancer: insights from a Delphi study in Spain

**DOI:** 10.3389/fonc.2025.1546019

**Published:** 2025-04-04

**Authors:** Enric Carcereny, Manuel Domine, Ana Laura Ortega Granados

**Affiliations:** ^1^ Medical Oncology Department, Catalan Institute of Oncology, Badalona, Spain; ^2^ Badalona Badalona Applied Research Group in Oncology (B·ARGO), Badalona, Spain; ^3^ Medical Oncology Department, Jiménez Díaz Foundation University Hospital- IIS- FJD, Madrid, Spain; ^4^ Medical Oncology Department, University Hospital of Jaén, Jaén, Spain

**Keywords:** metastatic lung cancer, cancer survivors, long-term survival, consensus, Delphi study

## Abstract

The improvement in survival rates in metastatic lung cancer (mLC) has increased the number of survivors’ special care needs. This study aimed to define and characterise these long-term survivors. A Delphi method with two successive rounds was conducted to reach a consensus (defined as an agreement ≥ 70%) on 56 items among 41 medical oncologists. The items included the definition of long-term survivors, their common characteristics, and oncological and non-oncological implications. The experts had an average age of 46 years, 53.7% were men, 90.2% attended for thoracic tumours, 40% had more than 15 years’ experience in mLC, and 56.1% of managing > 50 patients/month. Consensus reached 53.6% in the first round and 73.2% in the second. The definition of long-term survivors reached 58.3% consensus, defined as overall survival ≥ 3 years and/or progression-free survival ≥ 2 years. Identification of common features obtained 76.2% consensus on adenocarcinoma subtype of non-small-cell lung cancer, high PD-L1 expression, absence of brain metastasis, and fewer than two metastatic locations. Consensus was reached on specialized medical follow-up to detect immune-mediated toxicities and second neoplasms (87.8%), on pharmacological/non-pharmacological treatment for fatigue (82.9%) and sexual dysfunction (85.4%); and also on the importance of support for work and social adaptation (92.7%), integration of primary and hospital care (90.2%), implementation of quality-of-life programmes (92.7%) and electronic media (73.2%). This consensus identifies common characteristics and highlights relevant implications that should guide the follow-up and clinical management of these patients, ensuring better care and quality of life.

## Highlights

- Defined metastatic lung cancer (mLC) long-term survivors as those with overall survival ≥3 years or progression-free survival ≥2 years.
- Key features of long-term survivors include adenocarcinoma subtype, high PD-L1, limited metastases and treatment response.
- Specialized follow-up is vital for detecting immune toxicities and new cancers in mLC long-term survivors.
- Highlighted support for social/work adaptation and care integration as survivors’ needs.
- Emphasized quality-of-life programmes and electronic media as essential for long-term survivor care.

## Introduction

Lung cancer is one of the most commonly diagnosed cancers, and the leading cause of cancer deaths worldwide (1, 2). In Spain, the annual incidence in 2024 was estimated to be 32,768 cases; with a 5- year overall survival of 12% in men and 18% in woman (3). Lung cancer is diagnosed at a metastatic stage in half of patients, and these diagnostic delays contribute to the high 5-year mortality rates (4). Quality of life in patients with lung cancer is lower than in those with other malignancies, due to symptoms such as fatigue, dyspnoea, cough, blood in sputum, etc (5).

Lung cancer is a heterogenous malignancy, classified as non-small-cell lung cancer (NSCLC) in 85% of diagnoses, and as small-cell lung cancer (SCLC) in the remainder of cases. NSCLC includes adenocarcinoma, squamous-cell carcinoma and large cell carcinoma. Among the NSCLC subtypes, adenocarcinoma is the most common, while squamous-cell carcinoma has become less frequent, partly due to reduced smoking (1, 6). In recent years, treatment with anti-PD(L)-1 antibodies has improved long-term survival in NSCLC patients (7). Other biomarker tests used for therapeutic decision-making include EGFR mutations, ALK rearrangements or ROS1, NTRK, KRAS G12C, RET, MET, among others.

As a result, there are now more long-term survivors among lung cancer patients, who require specialised follow-up, coordinated between medical specialities and other healthcare services. The physical, psychological, social, occupational and emotional aspects of long-term survivors of lung cancer need to be characterised, investigated and treated by multidisciplinary and interdisciplinary teams. The specific healthcare problems of these patients should be identified and addressed in a structured way (8). However, there is no single criterion that identifies a patient as a long-term survivor. For early-stage NSCLC, long-term survival has been defined as OS ≥ 5 years (1, 9). For advanced-stage NSCLC and metastatic lung cancer, the definition varies between 18 months and 5 years (10–12). It is essential to define the characteristics of long-surviving patients, and the implications for patients and healthcare providers.

Several studies have investigated the characteristics of long-term survivors (12–15), and a consensus study has examined the optimization of the healthcare model for long-term cancer survivors, aiming to adapt it for use in Spain (16). However, to our knowledge, there is no consensus about the definition of a long-term survivor in advanced lung cancer, or the related clinical and non-clinical implications. The aims of this study were to define what is meant by long-survivor/long-responder, to establish what characteristics are common in these patients with metastatic lung cancer, and to analyse the oncological and non-oncological implications for patients and physicians.

## Materials and methods

### Study design

The study was led by a Scientific Committee consisting of three oncologists (the authors) involved in the treatment of patients with metastatic lung cancer, and who provide healthcare in public hospitals in Spain. Their role was to review the scientific literature and draft a questionnaire about long-term survival in patients with metastatic lung cancer. Statements/items included questions about both NSCLC and SCLC. They also validated and interpreted the statistical results from both rounds (intermediate and final analyses).

A modified Delphi study was carried out, involving two structured rounds of questions for obtaining consensus from geographically dispersed experts (Delphi panellists), and using online questionnaires. The Delphi method uses multiple rounds of controlled, anonymous feedback from experts via structured questionnaires (17, 18).

**Figure 1:**
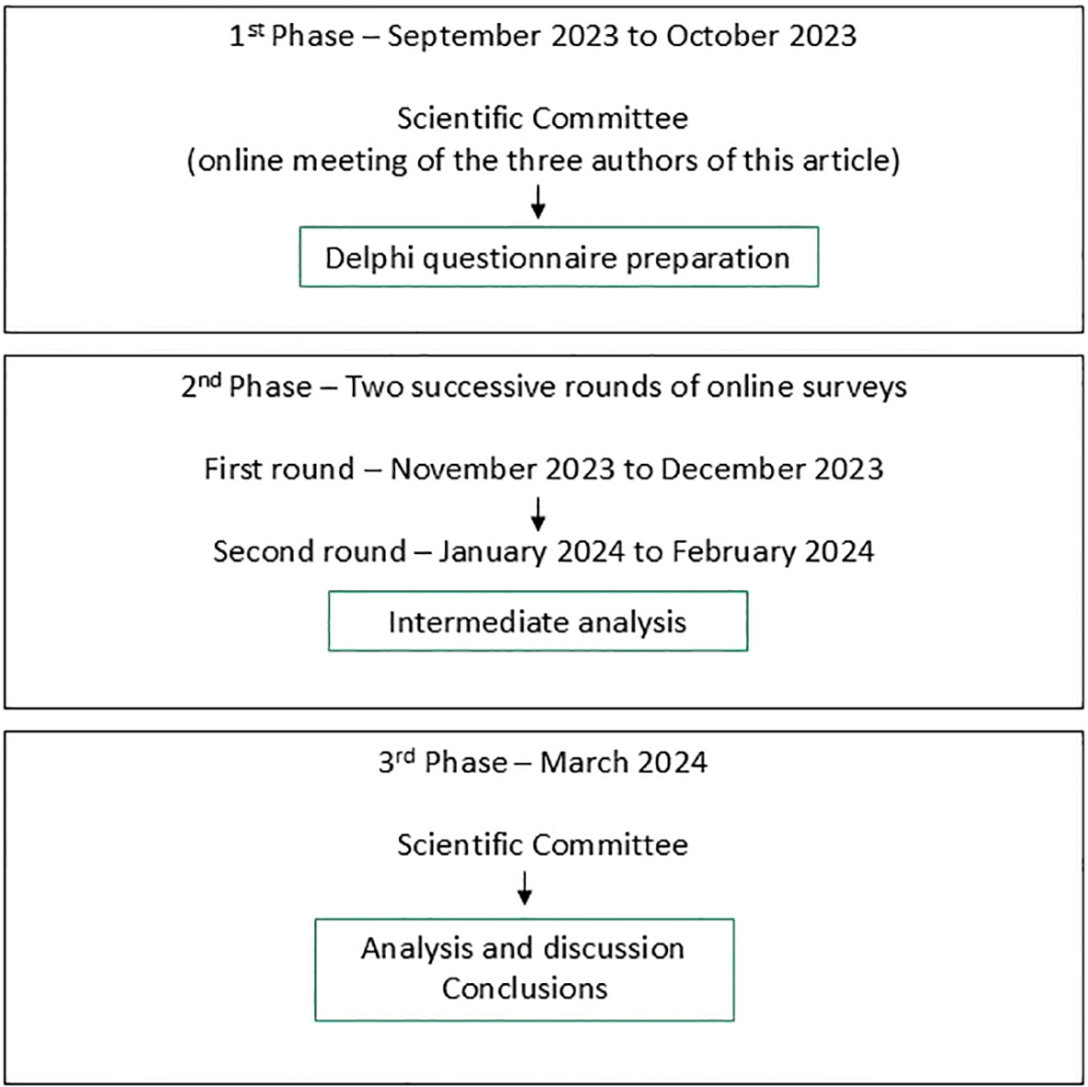
Phases of the Delphi process.

Potential panel experts were invited by the Spanish Lung Cancer Group (GECP: *Grupo Español de Cáncer de Pulmón*), on behalf of the Scientific Committee. Invitations were sent to 467 experts from the GECP member database. The selection criteria were: 1) Healthcare professionals specialized in medical oncology; 2) Active professionals with over 5 years of experience of treating patients with lung cancer; and 3) Professionals responsible for managing at least 10 patients with lung cancer per month. The study was performed in the three phases shown in **Figure 1**.

### Delphi questionnaires

Based on the discussions of the Scientific Committee, and in accordance with the evidence found about the subject, the Committee prepared a Delphi questionnaire consisting of 56 items grouped in 3 sections: 1) Definition of long-term survivors/long-term responders in metastatic lung cancer (12 items); 2) Common characteristics of long-term survivors/long-term responders in metastatic lung cancer (21 items); and 3) Oncological and non-oncological implications of long-term survival/long-term response in metastatic lung cancer (23 items).

The Delphi process involved two rounds of questionnaires using an online platform (encuesta.com). The answers to the questionnaires were gathered on a 9-point Likert scale, from 1 (completely disagree) to 9 (completely agree) (**Supplementary Table 1**).

The panellists provided feedback to each statement, individually and anonymously, based on their routine oncology practice and/or on the current clinical evidence.

### Two rounds of Delphi consensus

The first-round results were discussed by the Scientific Committee in an online meeting. Consensus for a statement was predefined as ≥ 70% responders scoring within the three-point range (1-3 or 7-9) containing the median. No statement reaching consensus in the first round was included in the next round of voting.

In the second round, an updated questionnaire comprising the statements without consensus was distributed for re-evaluation, and panellists could reconsider their initial opinion.

### Statistical analysis and interpretation of results

In a final meeting, the Scientific Committee discussed and interpreted the results. All statistical analyses were performed using the IBM SPSS Statistics 27. Nominal/ordinal variables were described with relative frequencies. For continuous variables, measures of central tendency and dispersion were computed. Statistical significance was determined using the chi-square test.

## Results

### Profile of Delphi panellists

**Figure 2:**
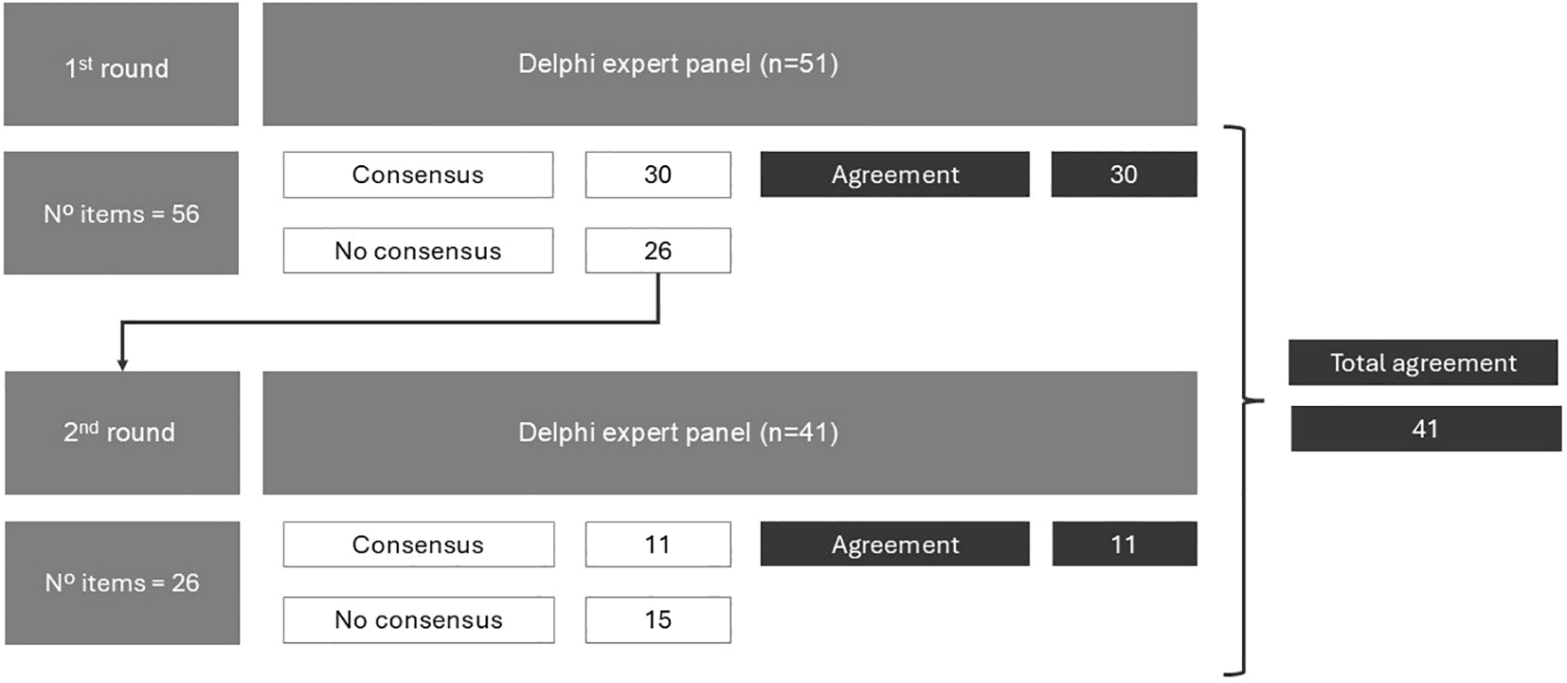
Results of the consensus in the two rounds of the Delphi process.

In the first round, 51 participants completed the questionnaire, and in the second round the number dropped to 41. Findings from the first round were recalculated and adjusted accordingly, based on these 41 experts (**Figure 2**). The characteristics of the experts are shown in **Supplementary Table 2**. Among the 41 experts, 53.7% were men, the average age was 46 (SD: 9) years old, 90.2% attended thoracic tumours (dealing with 1 or 2 oncological pathologies), 40% had more than 15 years of experience of treating patients with lung cancer, 56.1% saw more than 50 patients with lung cancer per month, and 51.2% worked in the most complex kind of hospital in Spain (Group 5).

### Consensus

In the first round, 56 items were proposed and there was consensus on 30 of them (53.6%). The remaining 26 items were subjected to further evaluation in the second round, in which consensus was achieved on 11 items. Thus, consensus was ultimately reached on 41 items (73.2%), and 15 items (26.8%) did not achieve consensus (**Figure 2**).

#### Definition of long-term survivor/long term-responder in metastatic lung cancer

There was consensus on 58.3% of the statements related to OS and progression-free survival (PFS) (**Table 1**). In metastatic lung cancer, a long-term survivor was defined as someone with OS of at least 3 years. Additionally, it was established that PFS of at least 2 years also indicates a long-term survivor in this disease. Furthermore, a positive correlation between PFS and the status of being a long-term survivor was recognised.

**Table 1:** Results of the two rounds of the Delphi process in statements about the definition of long-term survivor/long-term responder in metastatic lung cancer.

Statement/Item	Round	Disagree (score 1-3)	Undetermined (score 4-6)	Agree (score 7-9)	Consensus
Overall survival					
A patient with NSCLC is considered a long-term survivor when they reach an OS of at least 5 years from the cancer diagnosis.	1	4.9%	4.9%	90.2%	Yes
A patient with SCLC is considered a long-term survivor when they reach a 3-year OS from the cancer diagnosis.	2	4.9%	17.1%	78%	Yes
A patient with NSCLC is considered a long-term survivor when they reach a 3-year OS from the cancer diagnosis.	2	7.3%	14.6%	78%	Yes
A patient with SCLC is considered a long-term survivor when they reach a 5-year OS from the cancer diagnosis.	1	14.6%	14.6%	70.7%	Yes
A patient with SCLC is considered a long-term survivor when they achieve an OS of at least 2 years from the cancer diagnosis.	2	7.3%	26.8%	65.9%	No
A patient with NSCLC is considered a long-term survivor when they achieve a 2-year OS from the cancer diagnosis.	2	22%	48.8%	29.3%	No
Progression-free survival					
There is a positive correlation between PFS and being a long-term survivor in advanced lung cancer.	2	0%	12.2%	87.8%	Yes
A patient with advanced SCLC can be considered a long-term survivor when they achieve a PFS of at least 2 years from the start of treatment.	2	2.4%	19.5%	78%	Yes
A patient with advanced NSCLC can be considered a long-term survivor when they achieve a PFS of at least 2 years from the start of treatment.	2	4.9%	24.4%	70.7%	Yes
There is a positive correlation between treatment response and being a long-term survivor in advanced lung cancer.	2	2.4%	46.3%	51.2%	No
A patient with advanced SCLC can be considered a long-term survivor when they achieve a PFS of at least 1 year from the start of treatment.	2	29.3%	53.7%	17.1%	No
A patient with advanced NSCLC can be considered a long-term survivor when they achieve a PFS of at least 1 year from the start of treatment.	2	53.7%	31.7%	14.6%	No

NSCLC, non-small-cell lung cancer; OS, overall survival; PFS, progression-free survival; SCLC, small- cell lung cancer.

Consensus was not reached for the statements regarding SCLC or NSCLC and OS of at least 2 years, or a PFS of at least 1 year from the start of treatment. Additionally, there was no consensus on a positive correlation between treatment response and long-term survival.

#### Common characteristics of long-term survivors/long-term responders in metastatic lung cancer

There was consensus on 76.2% of the statements about the common characteristics of long-term survivors/long-term responders (**Table 2**), which were: oligometastatic NSCLC, adenocarcinoma subtype, with high PD-L1 expression (≥ 50%), without brain metastases or with tumour with less than 2 locations. Related to biomarkers and molecular targets, characteristics included the identification of mutations such as *ROS1*, *ALK*, *EGFR*, and high expression of PD-L1 (≥ 50%). Other characteristics were that the response to treatment was partial or complete, immunotherapy was continued for more than 2 years, the patient had been diagnosed in the last decade, had few comorbidities and a healthy lifestyle. The statement “There is a higher probability of being a long-term survivor in patients with advanced lung cancer with a stable caregiver (social and/or family support)” did not quite reach consensus (68.3%).

**Table 2:** Results of the two rounds of the Delphi process in statements about the common characteristics in long-term survivor/long-term responder in metastatic lung cancer.

Statement/Item	Round	Disagree (score 1-3)	Undetermined (score 4-6)	Agree (score 7-9)	Consensus
Tumour					
There is a higher probability of being a long-term survivor among patients with advanced NSCLC of the adenocarcinoma subtype than among patients with the squamous subtype.	1	0%	2.4%	97.6%	Yes
There is a higher probability of being a long-term survivor among patients with NSCLC than among patients with SCLC.	1	0%	2.4%	97.6%	Yes
There is a higher probability of being a long-term survivor among patients with oligometastatic advanced lung cancer, especially when combined with local treatments targeting metastases.	1	0%	2.4%	97.6%	Yes
There is a higher probability of being a long-term survivor among patients with advanced NSCLC and high PD-L1 expression (≥ 50%).	1	2.4%	14.6%	82.9%	Yes
The presence of brain metastases is associated with a lower probability of being a long-term survivor among patients with advanced lung cancer.	1	2.4%	17.1%	80.5%	Yes
The presence of metastases in more than two locations is associated with a lower probability of being a long-term survivor among patients with advanced lung cancer.	2	2.4%	22%	75.6%	Yes
There is a higher probability of being a long-term survivor among patients with advanced NSCLC and a high TMB due to increased response to ICI therapies.	2	4.9%	78%	17.1%	No^a^
Biomarker and molecular target					
There is a higher probability of being a long-term survivor among patients with advanced lung cancer and mutations in the *ALK* molecular target	1	0%	0%	100%	Yes
There is a higher probability of being a long-term survivor among patients with advanced lung cancer and mutations in the *EGFR* molecular target.	1	0%	2.4%	97.6%	Yes
There is a higher probability of being a long-term survivor among patients with advanced lung cancer and mutations in molecular targets than among those without them	1	0%	7.3%	92.7%	Yes
There is a higher probability of being a long-term survivor among patients with advanced lung cancer and mutations in the *ROS1* molecular target	1	2.4%	4.9%	92.7%	Yes
There is a higher probability of being a long-term survivor among patients with advanced lung cancer and mutations in other treatable molecular targets, excluding the ones mentioned earlier (*ROS1*, *ALK*, *EGFR*, PD-L1)^b^	2	2.4%	26.8%	70.7%	Yes
Treatment					
There is a higher probability of being a long-term survivor among patients with advanced lung cancer who have been treated with immunotherapy for a longer period (at least two years)	1	0%	9.8%	90.2%	Yes
There is a higher probability of being a long-term survivor among patients with advanced lung cancer who respond (completely or partially) to treatment than among those with disease stabilization	1	0%	14.6%	85.4%	Yes
Characteristics of the patient and family support					
There is a higher probability of being a long-term survivor among patients with advanced lung cancer diagnosed in the last decade	1	0%	4.9%	95.1%	Yes
There is a higher probability of being a long-term survivor among patients with advanced lung cancer with fewer comorbidities	1	2.4%	12.2%	85.4%	Yes
There is a higher probability of being a long-term survivor among patients with advanced lung cancer and a healthy lifestyle (diet, physical exercise, and absence of toxic substances)	2	0%	22%	78%	Yes
There is a higher probability of being a long-term survivor among patients with advanced lung cancer and a stable caregiver (social and/or family support)	2	4.9%	26.8%	68.3%	No
There is a higher probability of being a long-term survivor among patients with advanced lung cancer and a good socio-economic level	2	7.3%	58.5%	34.1%	No
There is a higher probability of being a long-term survivor among female patients with advanced lung cancer	2	4.9%	73.2%	22%	No
There is a higher probability of being a long-term survivor among patients with advanced lung cancer under 70 years of age	2	7.3%	78%	14.6%	No

ICI, immune checkpoint inhibitors; NSCLC, non-small-cell lung cancer; SCLC, small-cell lung cancer; TMB, tumour mutational burden.

a Statistically significant differences in response between rounds (p<0.05).

b Item rewritten between rounds to enhance its understandability.

#### Oncological and non-oncological implications of long-term survival/long-term response in metastatic lung cancer

There was consensus on 78.3% of the statements about oncological and non-oncological implications (**Table 3**). It was agreed that clinical follow-up was necessary to monitor the development of immune-mediated toxicities or secondary neoplasms, and that such monitoring should not be carried out by primary care. Concerning the clinical and emotional impact, there was agreement on the implementation of pharmacological and non-pharmacological measures to deal with fatigue and sexual dysfunction; nutritional guidelines, physical exercise, respiratory therapy, and medicines were recommended to enhance physical fitness and overall quality of life; and the provision of support and resources was also recommended to facilitate participation in work and social environments. Regarding healthcare resources, it was agreed that primary and specialized care should be integrated to optimise resources and coordination, to implement programmes for assessing quality of life, and to assess health outcomes to identify areas of concern among these patients. Electronic support for the selection of those patients who require urgent evaluation, and the review of evaluation criteria and funding for research projects, were also considered necessary.

**Table 3:** Results of the two rounds of the Delphi process about oncological and non-oncological implications.

Statement/Item	Round	Disagree (score 1-3)	Undetermined (score 4-6)	Agree (score 7-9)	Consensus
Medical follow-up					
Integrating primary and specialized care in the follow-up of long-term survivors of lung cancer is essential to optimise healthcare resources and ensure coordinated care	1	0	9.8%	90.2%	Yes
The emergence of second neoplasms in long-term survivors of advanced lung cancer can have a significant impact on patients, and good medical follow-up is necessary	1	2.4%	9.8%	87.8%	Yes
In patients with advanced lung cancer undergoing immunotherapy, and in long-term survivors, it is important to consider the possibility that immune-mediated toxicities may develop as a result of treatment	1	0%	12.2%	87.8%	Yes
Long-term follow-up in advanced lung cancer is associated with a lower risk of progression	2	2.4%	26.8%	70.7%	Yes
The follow-up of long-term survivors of lung cancer should be carried out in primary care	2	70.7%	22%	7.3%	Yes
The follow-up of long-term survivors of lung cancer should be carried out in specialized units for long-term survivors	2	4.9%	41.5%	53.7%	No
The medical procedures unrelated to lung neoplasia to be performed in a long-term survivor of lung cancer should be the same as in individuals without cancer^b^	2	58.5%	17.1%	24.4%	No
Approximately 25% of long-term survivors of lung cancer are lost to follow-up and do not continue survivorship care at the centre of treatment	2	46.3%	34.1%	19.5%	No^a^
The medical procedures unrelated to lung neoplasia to be performed in a long-term survivor of lung cancer should be the same as in other patients with advanced lung cancer^b^	2	48.8%	36.6%	14.6%	No
Clinical and emotional impact					
Returning to the workplace and the social environment can be a challenging process for long-term survivors of lung cancer, and it is important to provide support and resources to facilitate adaptation to a ‘new normal’ in their lives	1	0%	7.3%	92.7%	Yes
Physical exercise among survivors of advanced lung cancer has shown positive effects on physical condition, quality of life, anxiety, and self-esteem	1	0%	9.8%	90.2%	Yes
Psychological and cognitive care is essential for addressing the emotional and cognitive needs of long-term survivors of lung cancer and can help them cope with challenges related to their illness	1	0%	14.6%	85.4%	Yes
Sexual education, psychological support, or pharmacological measures are important means of addressing altered sexual function in long-term survivors of advanced lung cancer	1	0%	14.6%	85.4%	Yes
Relapse to smoking is a significant concern in long-term survivors of lung cancer, and should be addressed through specific prevention and support interventions	1	0%	17.1%	82.9%	Yes
In some cases, respiratory therapy and medication can improve physical fitness and can enable survivors to resume their normal daily activities	1	0%	17.1%	82.9%	Yes
Daily fatigue (asthenia or fatigue) is a common concern among long-term survivors of advanced lung cancer, and should be addressed by means of pharmacological, non-pharmacological, and/or psychosocial therapies	1	2.4%	14.6%	82.9%	Yes
Patients with advanced lung cancer experience psychological concerns such as living with uncertainty, fear of cancer progression, and anxiety about medical evaluations	1	2.4%	17.1%	80.5%	Yes
Implementing specific nutritional guidelines may help promote the health and well-being of long-term survivors of advanced lung cancer	1	2.4%	17.1%	80.5%	Yes
Cognitive decline is a concern among long-term survivors of advanced lung cancer treated with immunotherapy, and may affect their daily functioning and quality of life	2	12.2%	31.7%	56.1%	No
Healthcare resources					
Reviewing evaluation criteria and funding for research projects is essential to address the specific needs of long-term survivors of lung cancer	1	0%	2.4%	97.6%	Yes
Implementation of quality-of-life assessment programmes could be effective in identifying areas of concern among long-term survivors of advanced lung cancer	1	0%	7.3%	92.7%	Yes
Assessment of health outcomes could be effective in identifying areas of concern among long-term survivors of advanced lung cancer	1	2.4%	4.9%	92.7%	Yes
Implementing electronic decision-support for clinical selection of patients requiring urgent evaluation can improve medical care for long-term survivors of advanced lung cancer	2	0%	26.8%	73.2%	Yes

a Statistically significant differences in response between rounds (p<0.05).

b Item rewritten between rounds to enhance its understandability.

## Discussion

This is the first study that provides a definition of long-term survival in metastatic lung cancer. At the same time, this Delphi study has served to identify characteristics potentially associated with long-term survival in NSCLC and SCLC, and underscores the need to address both the oncological and the non-oncological aspects to enhance patient healthcare and quality of life.

Although previous literature suggests that a long-term survivor is defined as someone who survives for 2 years (12), this consensus opinion challenges that definition. In view of the results obtained here, long-term survival in metastatic lung cancer is defined as a minimum OS of 3 years. The results of this study also show that there is a positive correlation between PFS and long-term survivor status, and that when PFS is of at least 2 years, this can also be regarded as long-term survival.

Some studies have suggested that PFS of 1 year should be considered as long-term survival in patients with lung cancer (12, 19–21). Some authors consider that surviving for 5 years is rare for those with SCLC, and that 3 years would be enough to consider the patient a long-term survivor (22–24). Recent, positive clinical studies of new therapies show good outcomes among patients with advanced lung cancer at 2-3 years (21, 25, 26), and some recent studies show long-term survivors at 5 years (24, 27). These reports could explain the high neutrality of some participants about some statements in this consensus. There was no consensus about any correlation between treatment response and being a long-term survivor, probably because some experts considered stabilised patients as long-term survivors, whereas others did not.

Identifying common characteristics of long-term survivors of lung cancer is very important for shedding light on potential avenues for improving patient care and prognosis. It is very important to consider tumour characteristics. Patients with oligometastatic cancer and adenocarcinomas generally have longer survival (10, 28), and high expression of PD-L1 (≥ 50%) could be a predictor of long-term survival (12, 29). Although long-term survival is rare in lung cancer with brain metastases, some long-term survivors (≥ 3 years) among women and those with adenocarcinomas have been described (13).

There was no consensus about the statement that patients with advanced NSCLC with a high TMB are long-term survivors, because there is an increased response to immune checkpoint inhibitor (ICI) therapies. This lack of agreement could be explained by the fact that, although a high TMB has been found to be predictive of response to ICIs, this has not been validated prospectively (1). TMB has been associated with a better response to treatment (30), but there is not enough evidence to establish it as a biomarker for prolonged survival in lung cancer (12). Additionally, in many Spanish hospitals the limited infrastructure for TMB testing restricts its clinical use.

There was consensus about the importance of analysing molecular mutations that can be treated with targeted therapy (*ROS1*, *ALK* or *EGFR)*, and about the importance of a high expression of PD-L1(≥ 50%), for predicting prolonged survival among patients with metastatic lung cancer (1, 12). Some studies have explored the correlation between treatment response (partial or complete) and prolonged survival without conclusive results (31, 32). Although it has not been approached in this Delphi, it is interesting to note here that during immunotherapy, discontinuation of this treatment due to immune-related adverse events did not have a negative impact on the long-term benefits (33).

Concerning patient-related factors and their influence on long-term survival, one should consider the effect on long-term survival outcomes of being diagnosed within the last decade, and the significance of having fewer comorbidities (10) or of maintaining a healthy lifestyle when predicting prolonged survival (11, 12). There was no consensus about the effect of age, gender, having a caregiver or socio-economic status on long-term survival. Some studies have indicated a relationship between age, sex and survival – longer in younger patients and in women – and also that being married and having some form of social support increases the probability of survival (10, 11). The correlation between being married and longer survival (10, 34) could be explained by the better social support systems and better socio-economic status found among married patients or by the fact that married couples tend to adopt healthier lifestyles than unmarried people (35).

This consensus highlights the significance of recognising the clinical and non-clinical implications of long-term lung cancer survival that must be considered, and underscores the potential avenues for enhancing patient care and prognosis. The implications include medical follow-up, clinical and emotional impact, and healthcare resources. Regarding medical follow-up, immunotherapy drugs used in patients with lung cancer can cause various immune-mediated toxicities, such as pneumonitis, colitis, nephritis, and endocrinopathy. Long-term survivors may require ongoing medical monitoring to detect and manage these potential toxicities (9, 12) and the development of second neoplasms (36). It is recommended that follow-up care be overseen by specialized healthcare providers instead of relying solely on primary care physicians, thus ensuring comprehensive management of potential complications related to treatment.

There was no consensus on whether follow-up should be conducted in specialized units, possibly due to the absence of such units. This might suggest that specialized healthcare for these patients is important, but it does not necessarily mean that it is necessary to create specific units. Another explanation could be the lack of knowledge or training in this area and subjects. This kind of information is needed, and perhaps patient associations could intervene to obtain access to it.

No consensus was reached on whether medical procedures for patients with advanced lung cancer should follow those for other cancer patients or individuals without cancer. Despite being reformulated in the second round, these statements still lack consensus. Factors related to the patient, their neoplasm, or their treatment might suggest a need for different management approaches, but there is no evidence supporting these suggestions. Additionally, the definition of a long-term survivor remains unclear, and some items might not have been fully understood.

Consensus was not reached on the claim that 25% of patients are lost to follow-up, despite this being reported in cancer in general (37). Factors contributing to this lack of consensus include the absence of evidence on expected rates of follow-up loss, variations in individual clinician’s experiences, and hospital size.

In clinical and emotional impact, there are some unmet needs in several areas: physical, psychological, informational, spiritual, relational and daily living (15, 38, 39). Addressing the clinical and emotional well-being of long-term survivors requires a comprehensive, multidisciplinary approach. Both pharmacological and non-pharmacological interventions are essential in managing prevalent issues such as fatigue, aiming to improve overall quality of life for the individuals concerned. Cancer-related asthenia/fatigue ranks among the most prevalent and impactful challenges that affect patients’ quality of life, often persisting for months or years post-treatment. Therapeutic approaches should be individualised, incorporating the treatment of contributing factors, patient education, the implementation of general measures, pharmacological and non-pharmacological interventions, with physical exercise standing out among the latter (8, 15). In the relational context, sexual dysfunction associated with treatment also stands out. This disorder causes a significant reduction in quality of life, but it is not usually considered in oncological consultations. It is important to know its various causes and manifestations to be able to diagnose, evaluate and treat it correctly, in accordance with the best available evidence (8). Implementation of nutritional guidance, personalised exercise programmes, respiratory therapy, and appropriate medication can improve physical fitness and overall health outcomes (9, 40). In lung cancer survivors, pre-existence of lung function impairment may affect the treatment options. Respiratory therapy and medication can help in improving physical condition for daily activities (9). On a psychological level, increased cancer survival rates and life expectancy in recent decades have brought psychological and social adjustments. They can be manifested in a positive way, for example in enhanced problem-solving skills, strengthened relationships, and personal growth; but they may also lead to negative outcomes such as a fear of relapse, emotional distress, physical limitations, and difficulties in returning to work (8). Social and emotional support, together with access to resources, are crucial for helping long-term survivors adapt to changes in their work and social environments post-treatment (15).

Consensus was not reached about the impact of immunotherapy on the cognitive ability of long-term survivors and its implications for patients’ daily functioning and quality of life. Patients undergoing chemotherapy and/or radiotherapy often experience memory and concentration, and this affects their quality of life. Cognitive impairment is common, yet frequently underdiagnosed, with poorly understood underlying mechanisms and contributing factors. Affected cognitive domains include verbal memory, executive function, and processing speed. However, there is no consensus on which assessment tests should be conducted (8).

Efficient use of healthcare resources can be achieved through integrating primary and specialized care services, resulting in streamlined coordination and optimal patient management (41). The results of this Delphi study align with those of other consensus studies in emphasizing the importance of a multidisciplinary care model and improved coordination between primary and specialized healthcare. Additionally, they highlight the importance of addressing regional inequalities in healthcare resource allocation, which affect the effective implementation of the programmes identified in this study (42). Programmes focused on assessing quality of life and health outcomes can help to identify areas of concern and can then tailor interventions accordingly (43, 44). The use of electronic tools for decision-support – including tools to identify symptoms indicating cancer or disease progression – together with measures to minimize cognitive errors, can aid clinical decision-making and prioritize urgent evaluations of patients who require immediate attention (41). Reviewing assessment criteria and funding allocations for research projects can advance long-term survivorship care and improve patient outcomes (43). Tailoring protocols according to the specific needs and risks of lung cancer survivors is crucial. This includes early detection, addressing common problems, and considering patients’ characteristics and expectations in therapeutic decisions. The significance of emergency referral pathways, incorporating social workers and palliative care specialists, and recognizing psycho-oncology’s role in improving quality of life is underscored (42).

This study has several strengths. First, it provides insights into areas requiring greater emphasis, prioritising points needing more attention. Secondly, this is the first consensus that addresses the definition of long-term survivorship and its implications. And thirdly, as a Delphi study: 1) its anonymity encourages honesty in responses by eliminating the influence of dominant figures and reducing group bias, 2) it incorporates the expertise and knowledge of field experts, and 3) its iterative process of two rounds enables responses to be refined to promote greater consensus and more informed answers.

The study also has some limitations. First, those inherent in Delphi studies: 1) the lack of direct interaction with participants limits the ability to discuss and debate viewpoints among experts, potentially leading to a loss of valuable information that could have arisen from open debate (however, a scientific committee was in place to mitigate this limitation); 2) possible bias in structuring questions in the questionnaire, so the presentation of results could have influenced the direction of responses, and 3) although the questionnaire was developed from compiled scientific evidence, the responses are based on the subjective opinions of experts (which can be considered both a limitation and a strength). Secondly, the absence of patient participation. While physicians’ insights are valuable, incorporating patients’ perspectives would have allowed comparative analysis between the experiences of physicians and patients, potentially offering a richer understanding of long-term survival in metastatic lung cancer. This aspect is acknowledged, and possibility of addressing it in future is being considered. Thirdly, lack of objective data about the new situation of long-term survivors in lung cancer, and the absence of specific data from prospective studies, which may limit the conclusiveness of the findings and recommendations. Finally, the study was conducted in a single country, potentially limiting the generalization of the results to a global context. Expanding the scope to a multinational level could provide a more complete understanding of the subject. Future studies should incorporate patients’ data and consider multinational perspectives to further validate and enrich the study’s conclusions.

## Conclusions

According to the results obtained in this consensus study, long-term survival/long-term response in metastatic lung cancer is 3 years or more, and this has implications for treatment and prognostication. The definition of long-term survival as 2 years could become obsolete in view of the improved patient outcomes seen with targeted therapies and immunotherapy, and some criteria may need to be updated. Identification of factors such as PD-L1 expression, treatment response, and other tumoral characteristics may serve to identify patients with longer survival. The lack of consensus on specific management processes for long-term survivors, due to insufficient evidence, could be indicating the areas that require further research and development. There are innumerable implications, across various aspects of patients’ well-being, that affect survival, and this underlines the importance of comprehensive care approaches. Long-term follow-up and continuous monitoring are very important, regardless of whether they are conducted in specialized care settings or not, to ensure ongoing support and management. Future studies, particularly those incorporating patients’ data, will be crucial to further refine these findings and inform clinical practice, particularly in defining optimal care strategies for long-term survivors.

## Data Availability

The datasets presented in this article are not readily available because complete, aggregated results of the study are available upon request by the authors, but in no case can the individual results of the panellists be shared. Requests to access the datasets should be directed to ecarcereny@iconcologia.net.
