# Supplementary material for: Defining long-term survivors in metastatic lung cancer: insights from a Delphi study in Spain
Source: Front Oncol. 2025 Apr 4;15:1546019. doi: 10.3389/fonc.2025.1546019 (PMC12005997; doi:10.3389/fonc.2025.1546019)
Supplement: Supplementary file 1 [file Table1.docx]

**Supplementary Material**

**Supplementary Table 1** Level of agreement or disagreement of answers according to a 9-point Likert scale

| **Scale point** | **Degree of agreement/ disagreement** | **Responder’s tendency** |
| --- | --- | --- |
| 1 | Completely disagree | Strongly disagrees with the statement |
| 2 | Mainly disagree | Tends towards disagreement with the statement |
| 3 | Partly disagree | Has a slight tendency towards disagreement |
| 4 | Neutral | Neither agrees nor disagrees with the statement, but has a slight tendency towards disagreement |
| 5 | Neutral | Remains impartial or neutral towards the statement |
| 6 | Neutral | Remains impartial or neutral towards the statement, but has a slight tendency towards agreement |
| 7 | Partly agree | Strongly agrees with the statement |
| 8 | Mainly agree | Definitely agrees with the statement |
| 9 | Completely agree | Completely agrees with the statement |

**Supplementary Table 2** Demographics of the Delphi panellists

| **Variable** | **N (%)** |
| --- | --- |
| Sex  Woman  Man | 19 (46.3)  22 (53.7) |
| Specialization within medical oncology  Thoracic tumours (1-2 oncological pathologies)  Not focused on any type of tumour (≥ 3 oncological pathologies) | 37 (90.2)  4 (9.8) |
| Years of experience of visiting patients with lung cancer  5-10  11-15  16-20  > 20 | 16 (39)  9 (22)  6 (14.6)  10 (24.4) |
| Number of patients with lung cancer visited per month  10-15  26-50  > 50 | 2 (4.9)  16 (39)  23 (56.1) |
| Size and type of hospital  Group 1  Group 2  Group 3  Group 4  Group 5 | 1 (2.4)  1 (2.4)  9 (22)  9 (22)  21 (51.2) |

Group 1: District hospital, with less than 150 beds on average, and low complexity. Group 2: Basic general hospital, medium-sized with less than 200 beds, somewhat higher complexity than group 1. Group 3: Area hospital, medium-sized, with about 500 beds, and medium complexity. Group 4: Large hospital, heterogeneous in resources, size, and activity, with high teaching intensity and high complexity. Group 5: Hospital with a large structure and high activity, with comprehensive range of services; including large hospital complexes.
